# Comparison of Antibacterial Activities of ProRoot MTA, OrthoMTA, and RetroMTA Against Three Anaerobic Endodontic Bacteria

**Published:** 2018-09

**Authors:** Sedigheh Khedmat, Mohammad Aminipor, Maryam Pourhajibagher, Mohammad Javad Kharazifar, Abbas Bahador

**Affiliations:** 1 Professor, Dental Research Center, Dentistry Research Institute, Tehran University of Medical Sciences, Tehran, Iran; Department of Endodontics, School of Dentistry, Tehran University of Medical Sciences, Tehran, Iran; 2 Dentist, School of Dentistry, Tehran University of Medical Sciences, Tehran, Iran; 3 Researcher, Dental Research Center, Dentistry Research Institute, Tehran University of Medical Sciences, Tehran, Iran; 4 Statistical Advisor, Dental Research Center, Tehran University of Medical Sciences, Tehran, Iran; 5 Associate Professor, Department of Microbiology, School of Medicine, Tehran University of Medical Sciences, Tehran, Iran

**Keywords:** Bacterial Sensitivity Test, Mineral Trioxide Aggregate, OrthoMTA, ProRootMTA, RetroMTA

## Abstract

**Objectives:**

The aim of this study was to assess the antibacterial activities of OrthoMTA, RetroMTA, and ProRoot MTA against *Fusobacterium nucleatum* (*Fn*), *Porphyromonas gingivalis* (*Pg*), and *Prevotella intermedia* (*Pi*)*.*

**Materials and Methods:**

Each material was mixed on a glass slab using a spatula and was placed in columns containing the filter membrane of the modified membrane-enclosed immersion test (MEIT) system. The materials were sterilized after setting. The columns containing the sterilized test materials were placed in microcentrifuge tubes containing 500 μl of bacterial suspension. The systems were then incubated at 37°C under anaerobic conditions. After 72 hours, the bacterial growth and concentration (colony-forming unit (CFU)/ml) were assessed. The results were analyzed using one-way analysis of variance (ANOVA) and Tukey's post-hoc test in SPSS 22 software. In all analyses, the differences were considered significant at P<0.05.

**Results:**

OrthoMTA had the highest antibacterial activity against *Pi*. The mean number of CFU/ml of *Fn* in the presence of ProRoot MTA and RetroMTA was significantly lower than that in positive controls. There were significant differences between the antibacterial activities of ProRoot MTA and OrthoMTA against *Pg* compared to positive controls*.*

**Conclusions:**

ProRoot MTA, OrthoMTA, and RetroMTA had similar antibacterial activities against the three evaluated anaerobic endodontic bacteria, except RetroMTA against *Pg*.

## INTRODUCTION

Microorganisms have an essential role in exacerbation and improvement of pulpal and periapical diseases as well as the failure of endodontic treatment. Therefore, the eradication of microorganisms from the root canal system in endodontic treatment and prevention of bacterial entry into the root canal system during restorative treatment are the main factors for a successful clinical outcome [1–3]. Consequently, an ideal dental material, in addition to biocompatibility and sealing ability, should also have antibacterial effects [4].

Mineral trioxide aggregate (MTA) is the material of choice for repairing root perforations and various endodontic procedures such as root-end filling, pulp capping, pulpotomy, and apexification [5]. MTA has superior properties including biocompatibility, sealability, and bioactivity. However, the main drawbacks of MTA are long setting time, difficulty in handling, and tooth discoloration [6,7].

A new type of MTA (BioMTA, Seoul, Republic of Korea) has been proposed for use in various endodontic procedures as an alternative to ProRoot MTA. Two types of BioMTA include OrthoMTA and RetroMTA. According to the manufacturer, the setting time of RetroMTA is 150 seconds, and it causes no discoloration even after blood contamination. OrthoMTA is easy to handle using the OrthoMTA carrier. The setting time of OrthoMTA is 180 seconds [8].

Numerous studies have reported the antibacterial activity of MTA against microorganisms associated with endodontic disease, but the results are controversial [5,9–12]. Limited information is available on the comparative antibacterial activity of BioMTA against some of the principal bacteria involved in endodonticperiodontal infections including *Fusobacterium nucleatum* (*Fn*), *Porphyromonas gingivalis* (*Pg*), and *Prevotella intermedia* (*Pi*) [13].

The purpose of this study was to assess the antibacterial activities of OrthoMTA, RetroMTA, and ProRoot MTA against *Fn*, *Pg*, and *Pi.*

## MATERIALS AND METHODS

Lyophilized *Fn* (ATCC 25586), *Pg* (ATCC 33277), and *Pi* (ATCC 49046) cultures (Rayen Biotechnology Co., Ltd., Tehran, Iran) were rehydrated in Brain Heart Infusion (BHI) broth (Merck KGaA, Darmstadt, Germany) supplemented with hemin (5μg/ml; Sigma-Aldrich, Steinheim, Germany) and vitamin K (1 μg/ml; Sigma-Aldrich, Steinheim, Germany) and incubated in an anaerobic atmosphere at 37°C for 48 hours.

The test materials, including ProRoot MTA (Dentsply Maillefer, Ballaigues, Switzerland), OrthoMTA (BioMTA, Seoul, Republic of Korea), and RetroMTA (BioMTA, Seoul, Republic of Korea) were prepared according to the manufacturers’ instructions.

Each material was mixed on a glass slab using a spatula. The discs of freshly mixed MTA paste (approximately 4 mm in diameter and 2 mm in height) were placed in columns containing the filter membrane of the modified membrane-enclosed immersion test (MEIT) system. The modified MEIT system consists of two parts: 1) A column containing the filter membrane to hold the test materials, and 2) A microcentrifuge tube. After setting, the materials in the columns were sterilized with 25-kGy gamma rays.

The MEIT assay has been suggested for measuring the antibacterial activity of any water-based material including MTA in liquid cultures [14]. The filter membrane prevents the scattering of the test MTA and formation of MTA slurry in the microcentrifuge tube. The direct physical interaction between the bacterial cells and the test MTA allows for the exchange of soluble compounds between the membrane-enclosed material and the test bacteria [14]. The columns containing the sterilized test materials were placed in microcentrifuge tubes containing 500 μl of bacterial suspension at a final concentration of 10^5^ colony-forming units (CFU)/ml. Positive controls included bacterial suspension in the modified MEIT system without ProRoot MTA or BioMTA. Columns containing ProRoot MTA or BioMTA without bacterial suspension served as negative controls.

The systems were then incubated at 37°C under anaerobic conditions. After 72 hours, the bacterial growth and concentration (CFU/ml) were assessed. All experiments were performed in triplicate, and antibacterial effects of OrthoMTA, RetroMTA, and ProRoot MTA against *Fn*, *Pg*, and *Pi* were reported as mean ± standard deviation (SD).

The results were analyzed using one-way analysis of variance (ANOVA) and Tukey's post-hoc test in SPSS 22 software (IBM Co., Chicago, IL, USA). In all analyses, the differences were considered significant at P<0.05.

## RESULTS

The antibacterial activities of the evaluated MTA materials against the tested bacteria were significant compared to positive controls, except RetroMTA against *Pg*. The mean numbers of CFU/ml of each bacterium in the presence of the tested materials are presented in Table 1.

**Table 1.** The mean and standard deviation (SD) of bacterial concentrations (colony-forming unit (CFU)/ml) in the presence of three types of mineral trioxide aggregate (MTA)

** Materials **	** Bacteria **	** Mean **	** SD **
Positive Controls	* Pi *	49.3×10 ^ 5 ^	1.5×10 ^ 5 ^
	* Pg *	49.3	1.5
	* Fn *	49.3	1.5
ProRootMTA	* Pi *	10.6×10 ^ 5 ^	4.1×10 ^ 5 ^
	* Pg *	37	5.6
	* Fn *	20.7	4.5
RetroMTA	* Pi *	83.3×10 ^ 3 ^	68×10 ^ 3 ^
	* Pg *	46	1.7
	* Fn *	17.3	2.5
OrthoMTA	* Pi *	0	0
	* Pg *	37	4.6
	* Fn *	30.7	1.5

Pi=Prevotella intermedia, Pg=Porphyromonas gingivalis, Fn=Fusobacterium nucleatum

There were significant differences between the antibacterial activities of ProRoot MTA and RetroMTA (P=0.003), ProRoot MTA and OrthoMTA (P=0.002), and RetroMTA and OrthoMTA (P=0.001) against *Pi.* OrthoMTA had the highest antibacterial activity against *Pi*, while the antibacterial activity of RetroMTA was higher than that of ProRoot MTA.

There were similar antibacterial activities for ProRoot MTA and OrthoMTA against *P*g, and they both had significant differences (P=0.02) with positive controls*.*

The antibacterial activity of RetroMTA against *P*g was similar to that of the positive control group. There were no significant differences between the antibacterial activities of ProRoot MTA and OrthoMTA against *Pg.*

The mean number of CFU/ml of *Fn* in the presence of ProRoot MTA, OrthoMTA, and RetroMTA was significantly lower compared to the positive control group (P<0.001). The antibacterial activities of ProRoot MTA and RetroMTA against *Fn* were higher than that of OrthoMTA.

## DISCUSSION

According to the findings of the present study, the tested MTA materials had similar antibacterial activities against three species of anaerobic bacteria. The only exception was the non-significant antibacterial activity of RetroMTA against *Pg* compared to the positive control group.

Conversely, OrthoMTA had the highest antibacterial activity against *Pi*. The concentration (CFU/ml) of this species in the presence of this biomaterial was zero (100% reduction). Differences in the antibacterial activities of the two types of BioMTA in this study are probably the result of differences in their structure and composition. The manufacturer claims that RetroMTA has low cytotoxicity as it contains no heavy metals [15]. Also, bismuth oxide as a radiopacifier in OrthoMTA is replaced by calcium zirconia complex in RetroMTA [16]. The hydraulic calcium zirconia complex in RetroMTA can change the chemical and physical properties of the cement [17].

Lee et al [18] reported that the cytotoxicity of OrthoMTA was significantly higher than that of ProRoot MTA. They concluded that the initial amount of various ions released from the materials are different.

Donyavi et al [19] compared the antibacterial activity of ProRoot MTA, RetroMTA, and OrthoMTA against some bacteria commonly involved in endodontic infections using an agar diffusion test. They reported that RetroMTA and OrthoMTA had antibacterial activities similar to that of ProRoot MTA against *Enterococcus faecalis* (*Ef*) and *Streptococcus mutans* (*S. mutans*)*.* In the present study, the antibacterial activities of OrthoMTA and RetroMTA against the tested bacteria were comparable to that of ProRoot MTA.

Kouchak Dezfouli et al [20] evaluated the cytotoxicity of RetroMTA, as a new root-end filling material, compared to ProRoot MTA and showed similar biocompatibility for these two root-end filling materials.

Endodontic diseases are polymicrobial and predominantly induced by strict anaerobic bacteria [1,21,22]. In this study, the antibacterial effects of OrthoMTA, RetroMTA, and ProRoot MTA against *Fn, Pg, and Pi* were assessed. The isolation rate of *Fn* in endodontic infections varies by up to 85%, followed by *Pg* and *Pi* (65% and 62%, respectively) [23].

The MEIT system was used in this study. This technique is suitable for measuring the antibacterial activity of any water-based material, including MTA, without contamination of aqueous media throughout test periods. The filter membrane (0.45 μm) prevents the scattering of the test MTA and formation of MTA slurry in the wells [14]. The direct physical interaction between the bacterial cells and three types of tested MTA allowed for the exchange of soluble compounds between the membrane-enclosed material and the tested bacteria. During sampling, no tested material slurry was observed in the culture in the microcentrifuge tubes, indicating that the tested material remained mostly above the membrane. The absence of MTA slurry in aqueous media throughout the 72-hour period in the present study supports the use of the MEIT assay for direct and correct assessment of the antibacterial activity of these materials. The dispersion of MTA in aqueous media may cause errors in bacterial counts. Komabayashi and Spångberg [24] showed that about 90% of MTA particles would not pass through the pores of a 0.45-μm membrane.

Considering the important role of microbial biofilm in root canal infections, the use of a bacterial suspension model can be a limitation of the MEIT assay in this study.

There are some controversies regarding the antimicrobial effect of ProRoot MTA under anaerobic conditions. Although the antibacterial effect of ProRoot MTA against anaerobic bacteria has been proven previously [25,26], some researchers have reported that ProRoot MTA has no antibacterial effect against anaerobic bacteria [27–29]. They explained that the production of reactive oxygen species (ROS), as a by-product of aerobic metabolism, is partly responsible for the antimicrobial effect of ProRoot MTA. Since this by-product is decreased under anaerobic conditions, it has been speculated that ProRoot MTA may not produce adequate ROS to destroy the bacterial DNA and inhibit certain anaerobic bacterial strains [27–29]. The antibacterial effect of ProRoot MTA against the tested anaerobic bacteria in the present study was similar to those reported by some previous studies [25,26].

The main components of MTA are tri-calcium and di-calcium silicates. The hydration of these constituents forms an alkaline calcium silicate gel. Hydroxide ions release from calcium hydroxide in a silicate matrix. High alkalinity resulted from hydroxide ions creates an unfavorable environment for microbial growth [30,31].

This mechanism might be an explanation for the antibacterial effect of MTA against the tested bacteria in this study.

In the present study, OrthoMTA and RetroMTA had an acceptable antimicrobial activity against the two principal bacteria (*Fn* and *Pi*) present in endodontic-periodontal infections. Therefore, they may be used as an alternative to ProRoot MTA. However, further studies are required to confirm these results.

## CONCLUSION

ProRoot MTA, OrthoMTA, and RetroMTA had similar antibacterial activities against the three evaluated anaerobic endodontic bacteria, except RetroMTA against *Pg*.
